# The Association between Alcohol Consumption and Serum Metabolites and the Modifying Effect of Smoking

**DOI:** 10.3390/nu11102331

**Published:** 2019-10-01

**Authors:** Julia Langenau, Heiner Boeing, Manuela M. Bergmann, Ute Nöthlings, Kolade Oluwagbemigun

**Affiliations:** 1 Nutritional Epidemiology, Department of Nutrition and Food Sciences, Rheinische Friedrich-Wilhelms-University Bonn, 53115 Bonn, Germany; noethlings@uni-bonn.de (U.N.); koluwagb@uni-bonn.de (K.O.); 2 German Institute of Human Nutrition Potsdam-Rehbrücke, Division of Epidemiology, 14558 Nuthetal, Germany; boeing@dife.de (H.B.); bergmann@dife.de (M.M.B.)

**Keywords:** alcohol, smoking, targeted metabolomics, metabolite patterns, lipid metabolites, amino acids, acylcarnitines

## Abstract

Alcohol consumption is an important lifestyle factor that is associated with several health conditions and a behavioral link with smoking is well established. Metabolic alterations after alcohol consumption have yet to be comprehensively investigated. We studied the association of alcohol consumption with metabolite patterns (MPs) among 2433 individuals from the European Prospective Investigation into Cancer and Nutrition (EPIC)-Potsdam Study, and a potential modification by smoking. Alcohol consumption was self-reported through dietary questionnaires and serum metabolites were measured by a targeted approach. The metabolites were summarized as MPs using the treelet transform analysis (TT). We fitted linear models with alcohol consumption continuously and in five categories. We stratified the continuously modelled alcohol consumption by smoking status. All models were adjusted for potential confounders. Among men, alcohol consumption was positively associated with six MPs and negatively associated with one MP. In women, alcohol consumption was inversely associated with one MP. Heavy consumers differed from other consumers with respect to the “Long and short chain acylcarnitines” MP. Our findings suggest that long and short chain acylcarnitines might play an important role in the adverse effects of heavy alcohol consumption on chronic diseases. The relations seem to depend on gender and smoking status.

## 1. Introduction

Alcohol consumption has been shown to be associated to several health outcomes [1,2,3]. Metabolic alterations is one of the underlying mechanisms by which alcohol consumption exerts its effect on health [4,5,6]. One approach to explore metabolic alterations is through an analysis of the metabolome, a global analysis of small molecules emerging during human metabolism. The approach of metabolomics holds promise for an improved understanding of the impact of alcohol consumption on metabolic alterations. Several factors, such as genetic factors, dietary factors, and lifestyle factors, have been consistently shown to influence the metabolome [7,8,9]. Importantly, heavy alcohol consumers warrant attention since heavy drinking is associated with chronic diseases such as cardiovascular disease (CVD) [10], cancer [11] and liver cirrhosis [12]. Alcohol and tobacco use act synergistically on the risk of several diseases [11]. Thus, smoking may modify the association between alcohol and metabolites, but this has been investigated in only a few studies [13,14]. Thus, more studies are warranted.

There have been four studies describing the association of alcohol consumption and serum metabolites using a targeted metabolomics approach [15,16,17,18]. All of these studies reported that self-reported alcohol consumption was primarily associated with sphingomyelins and phospholipids. Furthermore, one of the aforementioned studies reported that alcohol consumption was associated with specific acylcarnitines and amino acids [17]. All of these studies have investigated associations of alcohol consumption with single metabolites. These studies corrected for multiple comparisons, however, important alcohol-metabolite associations might have been missed due to the fact that multiple testing increases the number of false negatives [19]. In order not to miss associations, and more importantly, considering that metabolites, especially those in the same class, have a high degree of intercorrelation, summarizing metabolites as patterns of metabolites would be an alternative approach. Treelet transform analysis (TT) is a data-driven novel statistical approach that can be used to capture interrelationships among metabolites and enables a good interpretability of groups of metabolites or metabolite patterns (MPs) [20].

Due to the social and physiological gender differences in alcohol consumption [21], and gender differences in metabolic profiles [22], it is necessary to specify gender-specific analyses a priori. Some of the previous studies [15,17] conducted gender-specific analyses as a post-hoc decision. It is well-known that subgroup analyses which have been pre-specified before data are available would eliminate data selection [23].

The aim of the present study was to investigate the association between alcohol consumption and gender-specific MPs, as well as a potential modification of these associations by smoking.

## 2. Materials and Methods

### 2.1. Participants and Study Design

European Prospective Investigation into Cancer and Nutrition (EPIC)-Potsdam is a part of the EPIC study and consists of 27,548 participants recruited from Potsdam and adjacent communities from 1994 to 1998. Baseline information obtained included anthropometric measurements, personal interviews on lifestyle habits, medical history, and blood sampling [24]. Written informed consent was obtained from all study participants. The study was conducted in accordance with the Declaration of Helsinki. Ethics approval was given by the Ethics Committee of the State of Brandenburg, Germany, on November 7th, 1993. 

This current study comprises a subsample of EPIC-Potsdam and includes 2500 participants (974 men and 1526 women) who were randomly selected in 2005 from all participants of EPIC-Potsdam who had provided blood sample at baseline (*n* = 26,444) [25]. For the present analysis, participants of the sub-cohort with missing data on serum metabolite concentrations (*n* = 33) and outliers of the MP scores (*n* = 9) were excluded. Moreover, we excluded participants with alcohol consumption >300 g/d (*n* = 2) due to the assumption that these participants might have pathophysiological changes that also affect metabolism. Following the exclusion of 23 participants who were out of the age range of recruitment (35–66 years) into the EPIC-Potsdam study, 2433 individuals (1499 women: 35–66 years, and 934 men: 40–66 years of age) were left for the present analysis.

### 2.2. Assessment of Alcohol Consumption

Alcohol consumption was assessed at baseline using a validated, self-administered food frequency questionnaire (FFQ) [26] and calculated based on the reported number of glasses of alcoholic beverages consumed during the 12 months prior to recruitment. Using empirically derived definitions of alcohol beverage-specific standard drinks in Germany, the number of glasses of intake was converted to grams of alcohol per day (g/day).

### 2.3. Assessment of Cigarette Smoking

The baseline questionnaire asked detailed questions on the history of cigarette smoking, including the number of cigarettes smoked per day and the age at which the participants stopped smoking. Based on the latter variable we calculated the time since cessation for former smokers (in years).

### 2.4. Assessment of Diet and other Lifestyle Variables

Habitual diet of the participants was also assessed by the self-administered, semi-quantitative FFQ at baseline [26]. Overall, the FFQ included 158 food items (including beverages) where average portion size and frequency of consumption during the previous 12 months had to be reported. During the baseline examination, physical activity, medical history, and educational and occupational status were assessed using standardized questionnaires. Moreover, anthropometric measures (height, weight and waist circumference) were collected by study staff following standardized procedures [24].

### 2.5. Measurement of Serum Metabolites

Metabolites were measured in one-time collected baseline serum samples with the AbsolueIDQ p150 kit (BIOCRATES, Innsbruck, Austria) by flow injection analysis tandem mass spectrometry (FIA)-MS/MS [27] at the Genome Analysis Center (Helmholtz Zentrum München). The targeted approach collected information on 163 predefined metabolites. These include small polar metabolites such as acylcarnitines, amino acids, hexose (sum of six-carbon monosaccharides without distinction of isomers), and lipids such as choline-containing phospholipids (Lyso-PC, diacyl- and acyl-alkyl-phosphatidylcholines and sphingomyelins). Sample preparation and metabolite quantification of these samples has been described previously [28]. In brief, at first 10 µL of serum were pipetted onto filters with stable isotope-labeled internal standards on a 96-well plate. Then the plates were dried in nitrogen stream and a derivatization of the amino acids with 5% phenylisothiocyanat reagent was conducted. Next, the plates were dried again and the remaining metabolites were extracted by internal standards using 5 mM ammonium acetate in methanol. After centrifugation and filtration, the final extracts were diluted with MS running solvent. Analysis of final extracts was performed with an API4000 triple quadrupole mass spectrometer (ABSciex, Framingham, MA, USA). Multiple reaction monitoring in combination with internal standards were applied for the quantification of metabolites, and concentrations were calculated in ‘mM’ using the MetIQ software package (BIOCRATES, Innsbruck, Austria). Overall, 36 metabolites with high variance (in the upper 10%) or below their limit of detection were excluded from the present analysis. Thus, the final metabolite set consist of 127 metabolites (17 acylcarnitines, 14 amino acids; 1 hexose; 34 diacyl-phosphatidylcholines; 37 acyl-alkyl-phosphatidylcholines; 10 Lyso-PC; and 14 SM).

### 2.6. Statistical Analysis

Data analyses were stratified by gender. Basic characteristics for the overall sample and by gender are presented as the mean ± standard deviation or as interquartile range for continuous variables and as percentages for categorical variables. Differences between genders were calculated with the T-test or Kruskal Wallis test for continuous variables and chi-square test (or Fisher’s exact test) for the categorical variables.

### 2.7. Identification of Metabolite Patterns

We divided the 127 included serum metabolites into two groups; small polar metabolites (amino acids, fatty acids, and sugar compounds) and lipids (sphingomyelins and phosphatidylcholines). The TT was used to derive MPs by reducing the dimensions of metabolites in each group to a few components. Prior to the TT, we log-transformed all metabolites in both groups. Thus, TT was used to create the correlation matrix of 32 small polar metabolites and 95 lipid metabolites in both men and women. We selected a favorable range (3–9) of (Treelet) metabolite components or patterns. Next, three successive 10-fold cross-validations of each number of components in 5 and 10 Monte Carlo repetitions were performed. The cut-level with the highest frequency was chosen as the optimal cut-level, and its corresponding number of components was used for the TT. In three stability runs we evaluated the stability of components using the bootstrap method—80% bootstrap-samples of the original data with 100 replications. We named the components according to the metabolites contributing to high loadings. We computed component MP scores for each individual by summing the standardized metabolite concentrations weighted by their loadings, across all metabolites. Thus, the MP scores are standardized values. Individuals with a high metabolite pattern score have a higher level of the metabolites contributing to these patterns as compared to individuals with a lower score. TT was performed using Stata SE14 (Stata Statistical Software: Release 14. College Station, TX: StataCorp LP, USA).

### 2.8. Multivariable Adjusted Linear Regression

Based on literature, we selected covariates such as body mass index (BMI), age and physical activity that are related to both alcohol consumption and metabolites profiles [29,30,31,32,33,34,35,36,37,38,39,40,41,42,43]. The minimum adjustment set of covariates was derived by a directed acyclic graph (DAG). Thus, model 1 is the unadjusted model; model 2 was adjusted for age, waist circumference-predicted BMI, physical activity index and socioeconomic status; and model 3 was additionally adjusted for food items (eggs, dairy products, fish, meat), nutritional supplements, and medication. Furthermore, for women we additionally adjusted model 3 for contraceptives and hormone replacement therapy.

We fitted linear regression models for the association between alcohol consumption (measured as a continuous variable) and MP scores. We modelled the continuous variable per 12 g/day (one standard drink in Germany). Additionally, in order to differentiate heavy consumers from other groups, we modelled established categories of alcohol consumption [44]): light (>0 to ≤2 g/d (m); >0 to ≤1 g/d (w)), below recommended limit (below RL) (>2 to ≤24 g/d (m); >1 to ≤12 g/d (w)), light to moderate (>24 g/d to ≤60 g/d (m); >12 g/d to ≤30 g/d (w)) and heavy (>60 g/d (m); >30 g/d (w)). One-way analysis of variance (ANOVA) was used to test the overall association of the categorical alcohol variable with the MP scores. For the MPs where we found a significant association, we analyzed the pairwise mean difference in the MP scores using Tukey’s Honest significant difference test.

To investigate the modifying effect of smoking, we stratified the continuous alcohol regression models by smoking status (non-smoker; former smoker and current smoker) for those alcohol-metabolite associations in which we found significant results.

In sensitivity analysis, for the MPs scores that were not significantly associated with alcohol in the continuously modelled alcohol intake at *p* < 0.05, we investigated whether there was evidence of nonlinearity using second-order polynomial regression models. The results of this analysis are shown in Table S1 and Table S2. The model fit of a nonlinear model as compared to its linear model counterpart was assessed by ANOVA. The statistical analysis was performed with the open source software R (version 1.1.442).

## 3. Results

### 3.1. Characteristics of the Study Population

Demographic characteristics for the study population are presented in Table 1. The mean age of study participants was around 50 years, with men being slightly older than women (52 compared to 49 years). Men also had higher BMI and waist circumference, and likely to be more educated and full-time employed as compared to women. Almost all men reported having drunk alcohol in the year previous to enrolment. Approximately half drank alcohol that was “below the recommended limit” and around one third were “moderate” drinkers. As for men, almost all women drank alcohol in the year previous to enrolment. The majority of women drank alcohol that was “below the recommended limit” and nearly one-fifth of the women were “moderate” drinkers. The median alcohol consumption at enrolment for women and men was around 5 g/d and 20 g/d, respectively. Almost three-quarters of the women and more than half of men were never smokers and, consequently, more men than women were current smokers. CVD was more prevalent in men, while cancer was more prevalent in women.

### 3.2. Identification of Metabolite Patterns

For men, we identified three small polar MPs and six lipid MPs with cumulative explained variance of 64.82% and 47.67%, respectively. The small MPs were named “Amino acids, sugar and free and short chain acylcarnitines (AAs, SUG, ACs)”, “Long and short chain acylcarnitines (ACs I)” and “Medium and long chain acylcarnitines (ACs II)”, and the lipid patterns were named “Diacyl-glycerophosphocholines and acyl-alkyl-phosphatidylcholine I (diacyl PCs, acyl-alkyl PCs I)”, “Sphingomyelins (SMs)”, “Lyso-phosphatidylcholines (lysoPCs)”, “Diacyl-phosphatidylcholines (diacyl PCs)”, “Diacyl-phosphatidylcholines and acyl-alkyl-phosphatidylcholine II (diacyl PCs, acyl-alkyl PCs II)” and “Acyl-alkyl-phosphatidylcholine (acyl-alkyl PCs)”. The explained variance and stability of each MP are shown in Table S3 and Table S4.

For women, we identified four small polar MPs and three lipid MPs with cumulative explained variance of 67.04% and 66.64%, respectively. The small MPs were named “Amino acids, sugar and free and short chain acylcarnitines (AAs, SUG, ACs)”, “Long and short chain acylcarnitines (ACs I)”, “Medium and long chain acylcarnitines (ACs II)”, “Short and medium chain acylcarnitines (ACs III)” and the lipid patterns were named “Diacyl-, acyl-alkyl-, lyso- phosphatidylcholines and sphingomyelins (diacyl, acyl-alkyl, lysoPCs, SMs)”, “Diacyl- and acyl-alkyl-phosphatidylcholine (diacyl, acyl-alkyl PCs)” and “Acyl-alkyl- and lyso-phosphatidylcholine (acyl-alkyl, lysoPC)”. The explained variance and stability of each MP are shown in Table S5 and Table S6. All metabolites in both genders were positively loaded on their MPs.

### 3.3. Multivariable Analyses of the Association between Alcohol Consumption and Metabolite Patterns

**Figure 1:**
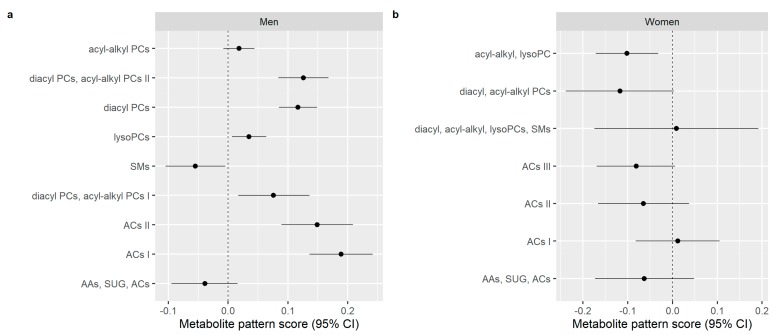
Associations between alcohol consumption in g/d and metabolite pattern scores at the time of recruitment (1994–1998) in EPIC-Potsdam; adjusted for age, sociodemographic and lifestyle factors, medications and chronic diseases-related medication. Changes in metabolite patterns scores with increased 12-g intake of alcohol per day in (**a**) men and (**b**) women; Abbreviation: AAs, SUG, ACs, Amino acids, sugar and free and short chain acylcarnitines, respectively; ACs I, Long and short chain acylcarnitines; ACs II, Medium and long chain acylcarnitines; ACs III, Short and medium chain acylcarnitines; acyl-alkyl, lysoPC, Acyl-alkyl- and lyso-phosphatidylcholine; diacyl, acyl-alkyl PCs, Diacyl- and acyl-alkyl-phosphatidylcholine; diacyl, acyl-alkyl, lysoPCs, SMs, Diacyl-, acyl-alkyl-, lyso- phosphatidylcholines and sphingomyelins.

After adjusting for age, sociodemographic and lifestyle factors, medications and chronic disease-related medication, a 12-g intake of alcohol per day was significantly associated with an increase in “ACs I” (*β* = 0.189, 95% CI: 0.136–0.242), “ACs II” (*β* = 0.149, 95% CI: 0.089–0.209), “diacyl, acyl-alkyl PCs I” (*β* = 0.076, 95% CI: 0.017–0.136), “diacyl PCs” (*β* = 0.117, 95% CI: 0.085–0.149), “lyso PCs” (*β* = 0.035, 95% CI: 0.006–0.064), “diacyl PCs, acyl-alkyl PCs II” (*β* = 0.126, 95% CI: 0.084–0.168) and a decrease in “SMs” (*β* = −0.055, 95% CI: −0.105–−0.005), in men (Figure 1a). Additionally, a 12-g intake of alcohol per day was significantly associated with a decrease in “SMs” (*β* = −0.055, 95% CI: −0.105–−0.005). Among women, a 12-g intake of alcohol per day was significantly associated with a decrease in “acyl-alkyl, lysoPC” (*β* = −0.102, 95% CI: −0.171–0.032) units (Figure 1b).

In men, except for “diacyl, acyl-alkyl PCs I”, the categorical alcohol variable was significantly associated with the previous MP scores from the linear model (“ACs I”, “ACs II”, “diacyl PCs”, “diacyl PCs, acyl-alkyl PCs II”, “lysoPCs” and “SM”). Among women, the categorical alcohol variable was significantly associated with the “acyl-alkyl, lysoPC”-MP. Table 2 represents the results of the multiple comparisons in all male participants. “Heavy” consumers had a significantly higher “ACs I” score compared to all other groups. They also have a higher “ACs II” score as compared to the “below RL”, “light” and “non-consumers” groups. With the exception of “light to moderate” consumers, “heavy” consumers have a higher “diacyl PCs” and “diacyl PCs, acyl-alkyl PCs II” score compared to almost all other consumer groups. In addition, they have a lower “SMs” score compared to “non-consumers” and “below RL”. There was no significant difference between alcohol groups for “lysoPCs” in men and “acyl-alkyl, lysoPC” in women. Due to the absence of evidence of a linear association, we fitted the second order polynomial regression for “AAs, SUG, ACs” and “acyl-alkyl PCs” in men, and among women, for “AAs, SUG, ACs”, “diacyl, acyl-alkyl PCs”, “diacyl, acyl-alkyl, lysoPCs, SMs”, “ACs I”, “ACs II”, “ACs III”. There was also no indication of a non-linear alcohol-metabolite pattern association for all aforementioned MP scores (Table S1 and Table S2). In fact, the nonlinear models were not significantly better when compared to their linear model counterparts.

We further examined the association between the continuously modelled alcohol consumption and MPs stratified by smoking status. The results are represented in Table 3. In men, the positive association of alcohol consumption with the “diacyl PCs, acyl-alkyl PCs I” MP was only consistent in never smokers, the inverse association of alcohol consumption with “SMs” MP was only consistent in current smokers, and the positive association of alcohol consumption with “ACs I”, “ACs II”, “lysoPC” and “diacyl PCs” MPs were consistent in both in never smokers and current smokers. In women, we found that the inverse association of alcohol consumption with “acyl-alkyl, lysoPC” MP was only consistent in current smokers.

## 4. Discussion

Our key finding was that alcohol consumption was associated with a number of metabolite patterns in men, an increase of the “ACs I”, “ACs II”, “diacyl, acyl-alkyl PCs I”, “diacyl PCs”, “lyso PCs” and “diacyl PCs, acyl-alkyl PCs II” MPs and a decrease in “SMs” MP. Among women, alcohol consumption was associated with a decrease of “acyl-alkyl, lysoPC” MP. Moreover, our results indicate that men consuming heavy alcohol differed from other consumers with respect to the “AC I” MP. Additionally, our study identified apparent smoking-related differences in the relation between alcohol consumption and MPs. These findings suggest that the impact of alcohol and smoking on health outcomes could be through their effect on changes in metabolite profiles.

Our result of the positive association between alcohol consumption and diacyl-, lyso- and acyl-alkyl PCs is consistent with previous studies that used targeted metabolomics [15,16,17,18]. Previous findings of a negative association between alcohol consumption and acyl-alkyl PC [15,16,17,18] could only be replicated in women. Moreover, in accordance with previous studies [15,16,17,18], we found a negative association between lysoPC (in women) and SMs (in men). In addition, our finding that alcohol consumption is positively associated with acylcarnitines is also in concordance with other results [15,17,18].

A possible explanation for our observed association between alcohol consumption and decrease of sphingomyelins is that alcohol stimulates sphingomyelinase activity [45,46,47]. Moreover, our observed association between alcohol consumption and decreased lysoPC might indicate less lipid remodeling in the membranes due to higher alcohol consumption [15]. Our results indicate that lipid profiles (e.g., diacyl-, lyso- and acyl-alkyl PCs and sphingomyelins) are particularly influenced by alcohol consumption. Since we know that lipids play an important role in energy metabolism it seems that alcohol consumption is associated with changes in energy metabolism.

Three studies investigated alcohol–metabolite associations with gender-specific differences using targeted metabolomics [15,17,18]. Our finding of alcohol–metabolite associations with gender-specific differences is consistent with the results from one of these studies [18]. In more detail, Jaremek et al. [18] identified specific profiles of 10 and 5 metabolites (sphingolipids and glycerophospholipids) in men and women, respectively, which separated participants with a consumption of <40 g/d and ≥40 g/d in men, and <20 g/d and ≥20 g/d in women. However, only three (for men) and two (for women) of the metabolites could be replicated in small samples. One explanation for these findings could be gender differences in alcohol metabolism due to body composition, genetic factors, gastric and hepatic alcohol dehydrogenase, and gastric absorption [48]. However, the authors [15] used a dichotomous categorization of alcohol consumption which might be suboptimal [49]. Furthermore, the reference group (<40 g/d and <20 g/d for men and women, respectively) included individuals exceeding the alcohol consumption recommended limit of 12 g/d for women and 24 g/d for men. It is possible that this categorization has an influence on the study results as there is probably an alcohol-specific metabolic dose-effect.

Our finding of men consuming heavy alcohol having increased metabolite pattern scores of “ACs I” compared to all other consumer groups suggests that heavy alcohol consumption might have distinctive metabolic effects. This may also be due to consumption of different or specific types of alcohol that may be peculiar to heavy consumers. Furthermore, it could be speculated that heavy consumers of alcohol already have altered pathophysiological changes in metabolism. One such pathophysiological change is mitochondrial dysfunction [50] and acylcarnitines which we observed in relation to heavy drinking might be potential biomarkers of mitochondrial dysfunction [51,52]. This suggests that long and short chain acylcarnitines might play a key role in the adverse effects of heavy alcohol consumption on chronic diseases such as the development and progression of atherosclerosis.

Interestingly the previously observed association between alcohol consumption and “ACs I”, “ACs II”, “lysoPC” and “diacyl PCs” are consistent in never and current smokers. The fact that these MPs in never and current smokers are linked to alcohol consumption indicates that these MPs are dependent on metabolite functions that are similar in both never and current smokers, one of which is the resting metabolic rate [53]. Since this association is no longer evident in former smokers, we speculate that either smoking cessation actually has metabolic effects or smoking cessation products may have masked these effects. The inverse association of alcohol consumption with lipid metabolites, “SMs” MP in male smokers, and “acyl-alkyl, lysoPC” MP in female smokers is noteworthy. Cigarette smoke and condensed tar contains free radicals, such as reactive oxygen species which promote peroxidation of lipids [54].

We explored non-linear associations between alcohol consumption and MPs. We found no evidence of nonlinearity for MP scores that were not significantly associated with alcohol in the linear model. This is consistent with the results obtained in [17]. In contrast, a study by Würtz et al. [55] found evidence of nonlinearity for alcohol–metabolite associations. It should, however, be noted that measured metabolites of the present study differ from the study by Würtz et al. [55]. Therefore, one can argue that observed differences in alcohol–metabolite associations are partly due to the differently measured metabolites.

Only one [17] of the current studies that investigated alcohol–metabolite associations adjusted the model for food items such as meat, fish and dairy products, despite evidence that these food items are linked to alcohol consumption and have a direct effect on serum metabolites [39,40,41,42,43]. We also captured the interrelationship among metabolites using a novel statistical approach, TT. The relevance of our approach in exploring MPs rather than single metabolites is that in other reports in which metabolites were explored individually, interpretation of these results was difficult. TT is a method that outperforms other dimension reduction methods in terms of interpretability of components [20,56]. Besides, unlike the hypothesis-based approaches, TT and other data-driven methods may be more optimal as they are based on the variation of the metabolites in specific populations. A strength of our study is the large sample size.

Our study focuses only on a limited subsample of metabolites; thus, we cannot rule out that other alcohol–MP associations might exist. Furthermore, the present study measured metabolites at a single-time point. However, metabolite concentrations do vary over time, therefore, future studies need to consider repeatedly measured metabolites. Another limitation of our study is that alcohol consumption was self-reported, which could introduce a potential measurement bias [57]. Future studies should consider complementing self-reported alcohol consumption with objective measures or biomarkers [58]. Biomarkers of alcohol consumption such as alcohol-ethanol that are detectable in noninvasively collected bio-samples like the urine [58]. Additionally, using second-order polynomial regression models might have underestimated nonlinear associations.

## 5. Conclusions

In conclusion, our findings suggest that the relation between alcohol consumption and groups of serum metabolites depends on gender and smoking status. Groups of metabolites such as long and short chain acylcarnitines might play an important role in the adverse effects of heavy alcohol consumption on chronic diseases. Alcohol consumption and smoking exhibit a synergistic effect on some metabolites. Generally, metabolomics is a powerful tool and provides additional information about metabolic pathways in alcohol metabolism. This approach has potential in various areas and helps us to improve our understanding of metabolic pathways in the relation of alcohol with health outcomes. Nevertheless, future studies are needed to replicate our findings, focus on the gender-specific differences, and investigate a wider range of metabolites.

## Figures and Tables

**Table 1:** Characteristics of the study population at the time of recruitment (1994–1998) in EPIC-Potsdam by gender.

Number of Participants	Total	Men	Women	*p*-Value
	2433	934	1499	
**Participant characteristics**				
Age^1^, years	50.46 (8.89)	52.61 (7.87)	49.13 (9.22)	<0.001
BMI^1^, kg/m^2^	26.14 (4.33)	26.80 (3.63)	25.73 (4.68)	<0.001
WC^1^, cm	85.90 (12.87)	94.18 (9.98)	80.74 (11.73)	<0.001
Education, university^2^	929 (38.2)	490 (52.5)	439 (29.3)	<0.001
Full time employment (≥35 h/week)^2^	1435 (59.0)	607 (65.0)	828 (55.2)	<0.001
Physically active, moderately inactive^2a^	956 (39.3)	348 (37.3)	608 (40.6)	0.157
Alcohol consumption^3^	8.54 (3.01, 20.6)	19.62 (8.92, 33.82)	5.16 (2.02, 10.76)	<0.001
**Alcohol consumption^2^**				<0.001
Non-Consumers^b^	72 (3.0)	31 (3.3)	41 (2.7)	
Light^c^	272 (11.2)	61 (6.5)	211 (14.0)	
Below recommended limit^d^	1403 (57.7)	479 (51.3)	924 (61.6)	
Light to moderate^e^	547 (22.5)	296 (31.7)	251 (16.7)	
Heavy^f^	139 5.7)	67 (7.2)	72 (4.8)	
**Smoking status*^2^***				<0.001
never smoker^g^	1627 (66.9)	553 (59.2)	1074 (71.6)	
former smoker^h^	352 (14.5)	175 (18.7)	177 (11.8)	
Current smoker	454 (18.7)	206 (22.1)	248 (16.5)	
**Number of cigarettes^2^**				<0.001
≤ 15, CPD	291 (64.1)	102 (49.5)	189 (76.2)	
16–24, CPD	112 (24.7)	63 (30.6)	49 (19.8)	
≥ 25, CPD	51 (11.2)	41 (19.9)	10 (4.0)	
**Prevalent Diseases^2^**				
Cancer	127 (5.2)	30 (3.2)	97 (6.5)	0.001
Stroke	27 (1.1)	18 (1.9)	9 (0.6)	0.005
Myocardial infarction	54 (2.2)	42 (4.5)	12 (0.8)	<0.001
**Medication^2^**				
Lipid-lowering Drugs	130 (5.3)	65 (6.9)	65 (4.3)	0.007
Antiphlogistika	5 (0.2)	4 (0.4)	1 (0.1)	0.149
Diuretics	57 (2.3)	21 (2.2)	36 (2.4)	0.916

Abbreviation: BMI, body mass index; cm, centimeter; CPD, cigarettes per day; kg, kilogram; m, meter; WC, waist circumstance; y, years; ^1^ Mean and standarddeviation in parentheses; ^2^ number and percentages; ^3^ median and interquartile range; ^a^ Cambridge physical activity index; ^b^ No consumption [no use of alcohol at enrolment/past 12 months]; ^c^ Light (>0 to ≤2 g/d (m); >0 to ≤1 g/d (w)); ^d^ below recommended limit (below RL) (>2 to ≤24 g/d (m); >1 to ≤12 g/d (w)); ^e^ light to moderate (>24 g/d to ≤60 g/d (m); >12 g/d to ≤30 g/d (w)); ^f^ heavy (>60 g/d (m); >30 g/d (w)); ^g^ Consisted of never smokers and ex-smoker who gave up smoking for ≥15years; ^h^ Ex-smoker who gave up smoking for ≤15 years.

**Table 2:** Mean score differences of male participants at the time of recruitment (1994–1998) in EPIC-Potsdam in six metabolite patterns.

Metabolite Patterns	Groups of Alcohol Consumers	Mean Score*
ACs I	Heavy	0.952a
	Light to moderate	0.372b
	Below RL	−0.098c
	Light	−0.317c
	Non-consumers	−0.283c
ACs II	Heavy	0.715a
	Light to moderate	0.227ab
	Below RL	−0.106bc
	Light	−0.604c
	Non-consumers	−0.208bc
SMs	Heavy	−0.240b
	Light to moderate	0.198ab
	Below RL	0.261a
	Light	0.066ab
	Non-consumers	0.592a
diacyl PCs	Heavy	0.541a
	Light to moderate	0.228a
	Below RL	−0.008b
	Light	−0.015b
	Non-consumers	−0.183b
diacyl PCs, acyl-alkyl PCs II	Heavy	0.461a
	Light to moderate	0.254a
	Below RL	−0.009b
	Light	−0.194b
	Non-consumers	−0.581b

Abbreviation: ACs I, Long and short chain acylcarnitines; ACs II, Medium and long chain acylcarnitines; diacyl PCs, Diacyl-phosphatidylcholines; diacyl PCs, acyl-alkyl PCs II, Diacyl-phosphatidylcholines and acyl-alkyl-phosphatidylcholine II; SMs, Sphingomyelins. Means followed by the same letter did not differ significantly (Tukey test, *p* > 0.05). Number of participants in each consumer group: non-consumers (*n* = 31), light (*n* = 61), below RL (*n* = 479), light to moderate (*n* = 269) and heavy consumers (*n* = 67); * mean of standard deviation score.

**Table 3:** Linear regression models stratified by smoking status in men and women at the time of recruitment (1994–1998) in EPIC-Potsdam.

Metabolite Patterns	Never Smoker		Former Smoker		Current Smoker	
	*β* (CI)	*p*-Value	*β* (CI)	*p*-Value	*β* (CI)	*p*-Value
**Men ^1^**						
ACs I	0.206 (0.137–0.276)	<0.001	0.094 (−0.052–0.240)	0.208	0.192 (0.072–0.312)	0.002
ACs II	0.169 (0.084–0.254)	<0.001	0.026 (−0.120–0.173)	0.726	0.141 (0.013–0.268)	0.032
diacyl PCs, acyl-alkyl PCs I	0.097 (0.015–0.178)	0.021	0.013 (−0.153–0.179)	0.877	0.037 (−0.077–0.151)	0.523
SMs	−0.035 (−0.101–0.032)	0.308	−0.057 (−0.198–0.084)	0.430	−0.137 (−0.252–−0.023)	0.020
lysoPC	0.043 (0.004–0.081)	0.030	−0.029 (−0.120–0.061)	0.525	0.075 (0.016–0.134)	0.014
diacyl PCs	0.127 (0.084–0.170)	<0.001	0.082 (−0.015–0.178)	0.100	0.119 (0.054–0.185)	<0.001
diacyl PCs, acyl-alkyl PCs II	0.153 (0.096–0.210)	<0.001	0.121 (0.005–0.238)	0.043	0.109 (0.026–0.192)	0.011
**Women ^2^**						
acyl-alkyl, lysoPC	−0.068 (−0.150–0.014)	0.104	−0.127 (−0.361–0.107)	0.289	−0.184 (−0.362–−0.007)	0.043

Abbreviation: ACs I, Long and short chain acylcarnitines; ACs II, Medium and long chain acylcarnitines; acyl-alkyl, lysoPC, Acyl-alkyl- and lyso-phosphatidylcholine; diacyl PCs, Diacyl-phosphatidylcholines; diacyl, acyl-alkyl PCs I, Diacyl-glycerophosphocholines and acyl-alkyl-phosphatidylcholine I; diacyl PCs, acyl-alkyl PCs II, Diacyl-phosphatidylcholines and acyl-alkyl-phosphatidylcholine II; lysoPCs, Lyso-phosphatidylcholines; SMs, Sphingomyelins. ^1^ Men: never smoker, *n* = 553; former smoker, *n* = 175; current smoker *n* = 206. ^2^ Women: never smoker, *n* = 1074; former smoker, *n* = 177; current smoker, *n* = 248.

## Supplementary Materials

The following are available online at https://www.mdpi.com/2072-6643/11/10/2331/s1, Table S1: Second order polynomial regression analysis of alcohol consumption and metabolite pattern scores in male participants, Table S2: Second order polynomial regression analysis of alcohol consumption and metabolite pattern scores in female participants, Table S3: Summary of small polar metabolite patterns of male participants, Table S4: Summary of lipid metabolite patterns of male participants, Table S5: Summary of small polar metabolite patterns of female participants, Table S6: Summary of lipid metabolite patterns of female participants.
