# Supplementary material for: The Association between Alcohol Consumption and Serum Metabolites and the Modifying Effect of Smoking
Source: Nutrients. 2019 Oct 1;11(10):2331. doi: 10.3390/nu11102331 (PMC6836136; doi:10.3390/nu11102331)
Supplement: Supplementary file 1 [file nutrients-11-02331-s001.pdf]

**Supplementary**

**The association between alcohol consumption and serum metabolites and the modifying effect of smoking**

**Table S1.** Second order polynomial regression analysis of alcohol consumption and metabolite pattern scores in male participants

| Metabolite pattern | Independent Variable[a] | β (CI) | p-Value |
|---|---|---|---|
| AAs, SUG, Acs | Alcohol consumption (12g/d) | -0.064<br>-0.190 – 0.063) | 0.322 |
| | Alcohol consumption (12g/d) ^2 | -0.000<br>(-0.000 – 0.000) | 0.673 |
| acyl-alkyl PCs | Alcohol consumption (12g/d) | 0.018<br>(-0.041 – 0.078) | 0.545 |
| | Alcohol consumption (12g/d) ^2 | -0.000<br>(-0.000 – 0.000) | 0.990 |

Abbreviation: AAs, SUG, Acs, Amino acids, sugar and free and short chain acylcarnitines; acyl-alkyl PCs, Acyl-alkyl-phosphatidylcholine; CI, confidence interval; adjusted for: age, body shape, physical activity index, socioeconomic status, eggs, dairy products, fish, meat, antihypertensive, diuretic, lipidlower, antiphlogistika, antibiotics, nutritional supplement, cancer; [a] Alcohol consumption is defined as alcohol consumption during the 12 months prior to recruitment per 12g/d; Alcohol consumption (12g/d) ^2 is the transformed variable of alcohol consumption we used for the polynomial regression analysis

**Table S2:** Second order polynomial regression analysis of alcohol consumption and metabolite pattern scores in female participants

| Metabolite pattern | Independent Variable[a] | β (CI) | p-Value |
|---|---|---|---|
| AAs, SUG, ACs | Alcohol consumption (12g/d) | -0.093 (-0.350 – 0.164) | 0.479 |
| | Alcohol consumption (12g/d) ^2 | 0.000 (-0.000 – 0.001) | 0.745 |
| ACs I | Alcohol consumption (12g/d) | 0.067 (-0.151 – 0.285) | 0.548 |
| | Alcohol consumption (12g/d) ^2 | -0.000 (-0.000 – 0.000) | 0.661 |
| ACs II | Alcohol consumption (12g/d) | -0.076 (-0.311 – 0.158) | 0.523 |
| | Alcohol consumption (12g/d) ^2 | 0.000 (-0.000 – 0.000) | 0.872 |
| ACs III | Alcohol consumption (12g/d) | -0.059 (-0.262 – 0.143) | 0.567 |
| | Alcohol consumption (12g/d) ^2 | -0.000 (-0.000 – 0.000) | 0.822 |
| diacyl, acyl-alkyl, lysoPCs, SMs | Alcohol consumption (12g/d) | 0.056 (-0.369 – 0.480) | 0.797 |
| | Alcohol consumption (12g/d) ^2 | -0.000 (-0.001 – 0.001) | 0.797 |
| diacyl, acyl-alkyl PCs | Alcohol consumption (12g/d) | -0.072 (-0.350 – 0.207) | 0.614 |
| | Alcohol consumption (12g/d) ^2 | -0.000 (-0.001 – 0.000) | 0.763 |

adjusted for: age, body shape, physical activity index, socioeconomic status, eggs, dairy products, fish, meat, antihypertensive, diuretic, lipidlower, antiphlogistika, antibiotics, nutritional supplement, cancer, contraceptives and hormone replacement therapy; Abbreviation: AAs, SUG, Acs, Amino acids, sugar and free and short chain acylcarnitines; acyl-alkyl PCs, Acyl-alkyl-phosphatidylcholine; CI, confidence interval; [a] Alcohol consumption is defined as alcohol consumption during the 12 months prior to recruitment per 12g/d; Alcohol consumption (12g/d) ^2 is the transformed variable of alcohol consumption we used for the polynomial regression analysis;

**Table S3.** Summary of small polar metabolite patterns of male participants

| Name metabolite pattern | Proportion of explained Variance in % | Number of metabolites | Metabolites (Loadings) | Stability in % |
|---|---|---|---|---|
| AAs, SUG, ACs | 41.28 | 17 | Acylcarnitines: C0(0.20), C3(0.20), Sugar: Hexose(0.23), Amino acids: Proline(0.23), Glycine(0.24), Serine(0.24), Threonine(0.24), Arginine(0.24), Tyrosine(0.24), Ornithine(0.25), Glutamine(0.25), Histidine(0.25), Methionine(0.25), Tryptophan(0.26), Phenylalanine(0.26), Valine(0.26), Leucine/Isoleucin(0.26) | 86 |
| ACs I | 14.03 | 6 | Acylcarnitines: C18:2(0.37), C18(0.39), C14:1(0.41), C2(0.42), C16(0.43), C18:1(0.43) | 89 |
| ACs II | 9.51 | 4 | Acylcarnitines: C16:2(0.47), C10(0.50), C14:2(0.51), C7-DC(0.51) | 92 |

Abbrevation: AAs, SUG, ACs, Amino acids, sugar and free and short chain acylcarnitines; ACs I, Long and short chain acylcarnitines; ACs II, Medium and long chain acylcarnitines; Acylcarnitines are abbreviated as Cx:y, where x and y are the the cumulative number of carbon atoms and number of double bonds, respectively.

**Table S4.** Summary of lipid metabolite patterns of male participants

| Name metabolite patterns | Proportion of explained Variance in % | Number of metabolites | Metabolites (Loadings) | Stability in % |
|---|---|---|---|---|
| diacyl PCs, acyl-alkyl PCs I | 25.04 | 27 | Phosphatidylcholine diacyl: C34:1(0.19), C34:2(0.19), C36:1(0.19), C36:2(0.19), C36:3(0.19), C36:4(0.19), C38:3(0.19), C38:4(0.19), C38:5(0.19), C40:4(0.19), C40:5(0.19), Phosphatidylcholine acyl-alkyl: C32:1(0.19), C34:1(0.19), C34:2(0.19), C34:3(0.19), C36:1(0.19), C36:2(0.19), C36:3(0.19), C38:2(0.19), C38:3(0.19), C38:4(0.19), C38:6(0.19), C40:3(0.19), C40:5(0.19), C36:4(0.20), C36:5(0.20), C38:5(0.20) | 60 |
| SMs | 10.67 | 11 | Hydroxysphingomyelin: C14:1(0.30), C16:1(0.30), C22:1(0.30), C22:2(0.30), C24:1(0.30), Sphingomyelin: C16:0(0.30), C16:1(0.30), C18:0(0.30), C18:1(0.30), C20:2 C24:0(0.30), C24:1(0.30) | 87 |
| lysoPCs | 3.95 | 4 | lysoPhosphatidylcholine acyl: C20:4(0.49), C18:1(0.50), C16:0(0.51), C18:0(0.51) | 87 |
| diacyl PCs | 3 | 3 | Phosphatidylcholine diacyl: C32:2(0.58), C34:3(0.58), C34:4(0.58) | 54 |
| diacyl PCs, acyl-alkyl PCs II | 2.98 | 3 | Phosphatidylcholine acyl-alkyl: C38:0(0.57), Phosphatidylcholine diacyl: C36:5(0.58), C36:6(0.58) | 88 |
| acyl-alkyl PCs | 2.03 | 2 | Phosphatidylcholine acyl-alkyl: C42:2(0.71), C42:3(0.71) | 62 |

Abbreviations: acyl-alkyl PCs, Acyl-alkyl-phosphatidylcholine; diacyl PCs, Diacyl-phosphatidylcholines; diacyl PCs, acyl-alkyl PCs I, Diacyl-glycerophosphocholines and acyl-alkyl-phosphatidylcholine I; diacyl PCs, acyl-alkyl PCs II, Diacyl-phosphatidylcholines and acyl-alkyl-phosphatidylcholine II; lysoPCs, Lyso-phosphatidylcholines; SMs, Sphingomyelins; The lipids are abbreviated as Cx:y, where x and y are the the cumulative number of carbon atoms and number of double bonds, respectively.

**Table S5.** Summary of small polar metabolite patterns of female participants

| Name metabolite patterns | Proportion of explained Variance in % | Number of metabolites | Metabolites (Loadings) | Stability in % |
|---|---|---|---|---|
| AAs, SUG, ACs | 37.44 | 17 | Acylcarnitines: C0(0.19), C3(0.19), Amino acids: Glycine(0.20), Threonine(0.23), Tyrosine(0.23), Ornithine(0.24), Proline(0.24), Serine(0.24), Arginine(0.25), Glutamine(0.26), Histidine(0.26), Methionine(0.26), Phenylalanine(0.27), Tryptophan(0.27), Valine(0.27), Leucine/Isoleucin(0.27), Sugar: Hexose(0.24), | 89 |
| ACs I | 13.16 | 6 | Acylcarnitines: C18(0.38), C18:2(0.38), C14:1(0.40), C2(0.42), C16(0.44), C18:1(0.44) | 91 |
| ACs II | 9.44 | 4 | Acylcarnitines: C16:2(0.48), C10(0.50), C14:2 (0.51), C7-DC(0.51) | 81 |
| ACs III | 7 | 3 | Acylcarnitines: C10(0.57), C3(0.58), C3-DC-M /C5-OH(0.58) | 92 |

Abbrevation: AAs, SUG, ACs, Amino acids, sugar and free and short chain acylcarnitines; ACs I, Long and short chain acylcarnitines; ACs II, Medium and long chain acylcarnitines; ACs III, Short and medium chain acylcarnitines; acylcarnitines are abbreviated as Cx:y, where x and y are the the cumulative number of carbon atoms and number of double bonds, respectively.

**Table S6.** Summary of lipid metabolite patterns of female participants

| Name metabolite patterns | Proportion of explained Variance in % | Number of metabolites | Metabolites (Loadings) | Stability in % |
|---|---|---|---|---|
| diacyl, acyl-alkyl, lysoPCs, SMs | 57.9 | 73 | Phosphatidylcholine diacyl: C 42:2(0.09), C30:0(0.10), C32:1(0.10), C36:0(0.10), C38:0(0.10), C42:5(0.10), C32:2(0.11), C34:3(0.11), C34:4(0.11), C36:5(0.11), C36:6(0.11), C40:2(0.11), C40:3(0.11), C40:5(0.11), C42:4(0.11), C28:1(0.12), C32:3(0.12), C34:1(0.12), C36:1(0.12), C36:3(0.12), C38:5(0.12), C38:6(0.12), C34:2(0.13), C36:2(0.13), C36:4(0.13), C38:3(0.13), C38:4(0.13), C40:4(0.13) Phosphatidylcholine acyl-alkyl: C40:1(0.10), C42:1(0.10), 38:0(0.11), C38:1(0.11), 32:1(0.12), C32:2(0.12), C34:0(0.12), C34:1(0.12), C34:2(0.12), C34:3(0.12), C36:1(0.12), C36:2(0.12), C36:3(0.12), C38:2(0.12), C38:3(0.12), C38:4(0.12), C38:6(0.12), C40:2(0.12), C40:3(0.12), C40:5(0.12), C40:6(0.12), C42:2(0.12), C42:3(0.12), C36:4(0.13), C36:5(0.13), C38:5(0.13) lysoPhosphatidylcholine: C16:0(0.11), C16:1(0.11), C17:0(0.11), C18:0(0.11), C18:1(0.11), C20:4(0.11), Sphingomyelin: C14:1(0.12), C16:1(0.12), C22:1(0.12), C22:2(0.12), C24:1(0.12), C26:0(0.10), C26:1(0.10), C16:0(0.12), C16:1(0.12), C18:0(0.12), C18:1(0.12), C24:0(0.12), C24:1(0.12) | 44 |
| diacyl, acyl-alkyl PCs | 6.87 | 8 | Phosphatidylcholine diacyl: C42:0(0.35), C42:1(0.35), Phosphatidylcholine acyl-alkyl: C42:4(0.33), C44:4(0.34), C40:4(0.35), C44:5(0.37), C44:6(0.37), C42:5(0.37) | 72 |
| acyl-alkyl, lysoPC | 1.87 | 2 | Phosphatidylcholine acyl-alkyl : C30:2(0.71), lysoPhosphatidylcholine: C28:1(0.71) | 71 |

**Abbreviations: acyl-alkyl, lysoPC, Acyl-alkyl- and lyso-phosphatidylcholine; diacyl, acyl-alkyl PCs, Diacyl- and acyl-alkyl-phosphatidylcholine; diacyl, acyl-alkyl, lysoPCs, SMs, Diacyl-, acyl-alkyl-, lyso- phosphatidylcholines and sphingomyelins; The lipids are abbreviated as Cx:y, where x and y are the the cumulative number of carbon atoms and number of double bonds, respectively.**
